# Tularemia: A Unique Presentation for a Rare Disease

**DOI:** 10.7759/cureus.84503

**Published:** 2025-05-20

**Authors:** Jake Allison, Jonathan Williamson, Heather Young, Zain Alamarat

**Affiliations:** 1 Pediatric Infectious Diseases, University of Arkansas for Medical Sciences, Little Rock, USA

**Keywords:** gentamicin, oculoglandular disease, oropharyngeal, oropharyngeal disease, ticks, tularemia

## Abstract

This report highlights an uncommon presentation of tularemia in a high-risk pediatric patient to increase awareness and broaden the differential diagnosis among clinicians. A previously healthy eight-year-old female presented to the emergency department multiple times within two weeks with nonspecific flu-like symptoms, worsening purulent conjunctival injection, fever, and left-sided facial swelling. She was initially diagnosed with several conditions, including corneal abrasion, preseptal cellulitis, and dacryocystitis, and was treated with antibiotics without symptom resolution. Upon admission, the patient showed signs of a severe infectious process with febrile illness, leukocytosis, and orbital involvement. A detailed history revealed high-risk exposures, including tick bites and animal contact. Tularemia testing was positive, and the patient was treated with a prolonged course of gentamicin, resulting in complete resolution of symptoms. Tularemia, caused by *Francisella tularensis*, can present with various symptoms and is often difficult to diagnose without high clinical suspicion. The case emphasizes the importance of considering rare diseases such as tularemia in endemic regions, especially when initial treatments fail.

## Introduction

This report presents the case of an eight-year-old female with multiple emergency department (ED) visits initially for a corneal abrasion with conjunctivitis that worsened to progressive, antibiotic-resistant preseptal cellulitis before eventually being diagnosed with oropharyngeal and Parinaud oculoglandular syndrome as a combined presentation of tularemia. Tularemia is a disease typically transmitted by arthropods or other infected animals carrying *Francisella tularensis* to humans. Cases have been reported in all the United States except Hawaii [1]. Most cases occur in the south-central U.S. and the Pacific Northwest during the summer months, as tick bites are responsible for most human infections [2]. Furthermore, tularemia incidence appears to be rising, with one study showing a 60% increase in incidence from 2011 to 2019 compared to the prior decade [3]. Tularemia is extremely transmissible, with some studies suggesting that as few as 10 organisms can produce infection [4]. The disease manifests in one of seven well-documented forms: ulceroglandular or glandular (the two most common forms), oculoglandular, oropharyngeal, intestinal, pneumonic, or typhoidal [3]. Due to the non-specific, “flu-like” nature of initial symptoms, diagnosis tends to be delayed, with a mean time of 19-41 days from symptom onset to serologic testing in studies [5-6]. If treatment is delayed, disease progression can result in serious complications including lymphadenitis requiring surgical debridement, dacryocystitis, conjunctival nodular ulcers [6-7], and even death. Our case represents a unique presentation given that review of current literature in the PubMed databases using keywords “Tularemia”, “oropharyngeal”, “oculoglandular”, and “children” revealed no reported incidences of a patient presenting with two of the clinical syndromes associated with *F. tularensis* infection. Our goal with this report is to increase recognition of this disease in endemic and non-endemic regions. We present this case for its crucial insight and to add to the medical acumen of physicians to allow improved early detection, early treatment, and decreased complications from tularemia infection.

This case was previously presented as an oral presentation at the 2024 Southern Society for Pediatric Research (SSPR) conference on February 22, 2024.

## Case presentation

A previously healthy eight-year-old female presented to the ED with fever, vomiting, and body aches, preceded by left eye pain and irritation. She was initially diagnosed with a corneal abrasion and prescribed erythromycin eye ointment to be applied four times daily for seven days with amoxicillin-clavulanic acid 45 mg/kg orally for 10 days. Two days later, she returned to the ED with worsening pain and redness around the left eye and was diagnosed with preseptal cellulitis. Her antibiotic was switched to clindamycin 10 mg/kg three times daily for 10 days, yet she returned the next day due to worsening left eye swelling and development of left neck swelling, at which time she was diagnosed with lymphadenitis and instructed to continue current treatment. Five days later, she again presented with continued worsening of her eye swelling that was becoming more localized. She was diagnosed with dacryocystitis and discharged without any change in management. Three days later, she was seen by her primary care physician due to persistence of symptoms, given one dose of intramuscular ceftriaxone, and sent to the ED for anticipated inpatient admission.

At the time of admission, she was ill-appearing, febrile to 38.2°Celsius, with left periorbital erythema and swelling plus overlying ecchymosis of the left nasolacrimal duct and limitation of left eye extraorbital motion. She was noted to have bilateral oropharyngeal exudate and posterior oropharyngeal erythema with multiple, large, mobile, tender submandibular and cervical lymph nodes on the left side (several with overlying erythema). She also had a diffuse, faint, macular rash across the body that spared the palms and soles.

Initial workup on admission to the hospital revealed leukocytosis, mild anemia, mild thrombocytosis, and an elevated C-reactive protein level (Table 1).

**Table 1:** Initial lab values at hospital admission

Lab	Value	Reference Range	Units
White blood cells	28,170	4,500-13,500	WBC per microliter
Hemoglobin	10.7	11.5-15.5	g/dL
Platelets	489,000	150,000-450,000	platelets per microliter
C-reactive protein	70	0-9.9	mg/L

**Figure 1:**
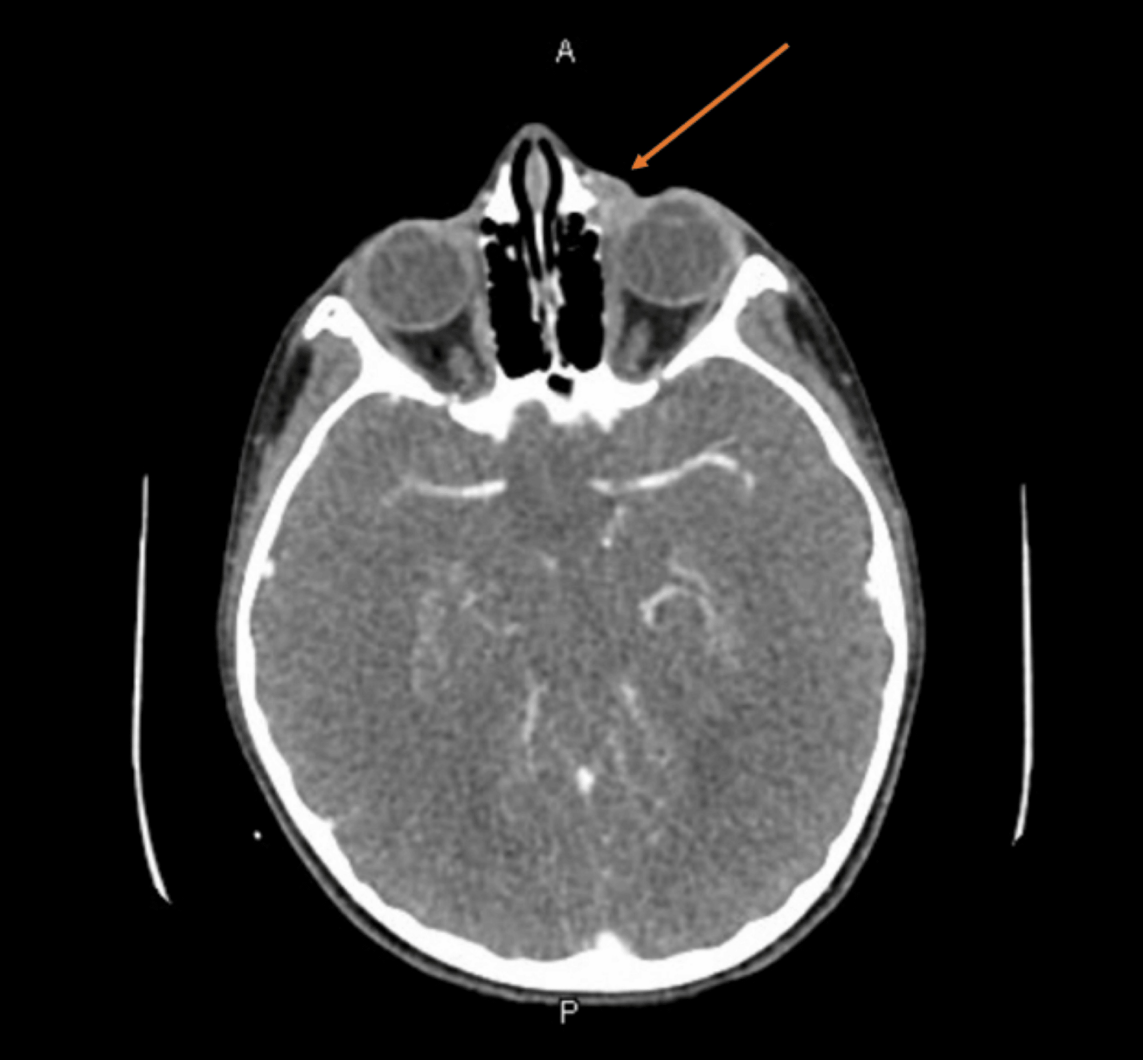
CT of the orbits with contrast The arrow points to the area of opacification commented on by radiology as dacryocystitis.

CT scan of the orbits with contrast revealed persistent findings of left dacryocystitis and no signs of post-septal extension or abscess (Figure 1).

**Figure 2:**
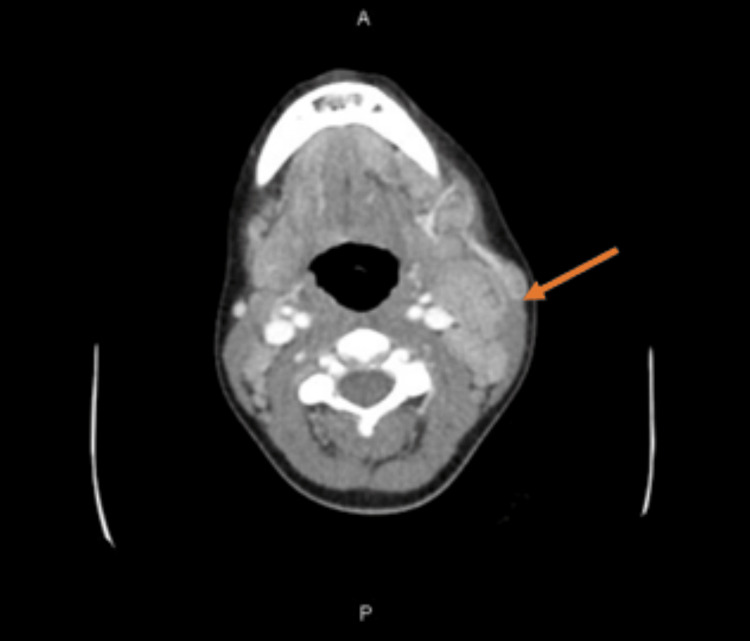
CT of the soft tissue neck with contrast The arrow points to the area of significant lymphadenopathy in the left cervical chain.

CT of soft tissue neck with contrast revealed adenoid and palatine tonsillar enlargement and left cervical adenopathy with signs of suppuration in several cervical nodes, and no signs of abscess (Figure 2).

She was started on vancomycin and doxycycline on admission. The infectious disease service was consulted and elicited further exposure history: she lived on a farm with cattle, sheep, cows, pigs, horses, dogs, and cats, her family made their own unpasteurized cheese and milk, she had a tick removed from her left ear on day eight of illness, and the family was notified that their new puppy had been playing with a dead rabbit just prior to the family picking them up from the breeder.

By hospital day 3, she had been afebrile with minimal improvement of swelling but down-trending inflammatory markers and was discharged on doxycycline 2.2 mg/kg twice daily to complete a 10-day course. Two days later, her tularemia testing returned positive with the values listed in Table 2.

**Table 2:** Tularemia serologies

Serology	Value	Reference Range	Units
Tularemia IgM	>100	<9	units/mL
Tularemia IgG	95	<9	units/mL
Tularemia Ab by agglutination	1 to 640	<1 to 20	NA

The family was contacted. They noted no new fevers but no improvement in the neck swelling, and she developed new oral ulcers. She was re-admitted, and her antibiotics were switched to intravenous gentamicin 9 mg/kg daily (titrated to this dosage based on trough levels obtained while inpatient).

Due to a good clinical response to the daily dosing of intravenous gentamicin, a peripherally inserted central catheter (PICC) line was placed, and she was discharged after a few days on gentamicin to complete a 10-day course of therapy. Follow-up visit with infectious disease four days after antibiotic completion and PICC line removal revealed resolution of disease. The family was called approximately 6 and 12 months after completion of therapy, who confirmed continued resolution of disease without recurrence.

## Discussion

Tularemia’s initial presentation often includes non-specific, flu-like symptoms, making the diagnosis difficult without a high degree of suspicion. Morbidity increases with delays in antibiotic therapy. Disease progression results in at least one of the syndromes discussed in this report. For prompt initiation of appropriate therapy, physicians should be cognizant of all forms of disease presentation, especially in a patient from an endemic area with significantly high-risk exposures.

Our patient had multiple presentations, with each of them having a different differential diagnosis. Initially, for corneal abrasion, the differential included trauma, foreign body, or viral infection such as herpes simplex. When diagnosed with preseptal cellulitis, other conditions such as orbital cellulitis or dacryocystitis, which could be viral or bacterial in nature, needed to be considered. When unilateral lymphadenopathy was added to her constellation of symptoms, *Bartonella henselae*, acute HIV, Brucellosis, Rocky Mountain spotted fever, and non-tuberculous mycobacterial infection needed to be considered, especially given her broad exposure history. Finally, with the development of oral and pharyngeal ulcers, adenovirus, enterovirus, more widespread herpes simplex, or Epstein-Barr virus were added to the differential diagnoses. As mentioned above, tularemia testing would provide her definitive diagnosis.

Tularemia is most often diagnosed by serology. A single elevated IgM level can take up to 14 days to develop; thus, after two weeks, a fourfold or more increase between acute and convalescent serum is diagnostic [8]. This patient’s serologies were obtained about two weeks into her illness. While PCR testing is an option and could potentially provide a faster diagnosis, it is not yet available at most hospitals or clinical laboratories [9]. Isolation of the organism in cysteine-enriched culture media is diagnostic, but if *F. tularensis* is suspected, then the laboratory personnel should be alerted immediately to take the necessary precautions [8].

Our patient was eventually diagnosed with Parinaud oculoglandular syndrome and oropharyngeal tularemia. Either of these syndromes is rare in and of themselves, and concomitant presentation of oculoglandular and oropharyngeal tularemia has not yet been reported, per our review of the literature. A study reviewing tularemia cases in an endemic area from 2009 to 2013 found that oculoglandular tularemia comprised only 2% of cases and oropharyngeal tularemia comprised only 4% of cases [5]. The most common presentations found in the study were typhoidal (50%) and ulceroglandular (24%) tularemia [5]. Another study of cases in the United States from 2006 to 2021 found that ulceroglandular disease accounted for 42% of cases, while oculoglandular and oropharyngeal disease accounted for only 2% and 1.5% of cases, respectively [9]. In their survey of tularemia cases across the United States, Wu et al. found that cases of oculoglandular and oropharyngeal tularemia had lower rates and longer times to appropriate antimicrobial treatment [9]. The study authors did not propose theories on why this occurs, but it is reasonable to assume that providers are less familiar with these presentations and therefore have a lower level of clinical suspicion.

The treatment of choice is an aminoglycoside, most commonly gentamicin, either three times daily or once daily, with the dose adjusted to achieve a desired peak of 5 mg/mL, due to the limited availability and toxicity of streptomycin [8,10]. A seven-day course may be sufficient in mild cases, but 10-14 days or longer is more commonly used for severe infection. Ciprofloxacin is an alternative for mild cases, but it is not approved by the U.S. Food and Drug Administration (FDA). Meningitis or endocarditis should be treated with both an aminoglycoside and either ciprofloxacin or doxycycline [8,11-12]. Chloramphenicol is another antibiotic that has been used with success but is not widely available and has significant toxicities. *Francisella tularensis* is not susceptible to beta-lactams [13].

While aminoglycosides, fluoroquinolones, and tetracyclines have activity against *F. tularensis*, the effectiveness of these antibiotics differs, with tetracyclines particularly having a high relapse rate. Prior to our patient’s diagnosis, she was treated with doxycycline, while testing for multiple tick-borne illnesses was pending. Tetracyclines are bacteriostatic, and, while common resistance in clinical isolates has not been reported, treatment typically requires prolonged courses (such as 21 days to fully treat) when compared with aminoglycosides [8]. Our patient did not have significant relief of her symptoms until gentamicin was started.

## Conclusions

Tularemia is a rare disease with multiple clinical presentations. The illness can have high morbidity if not treated with appropriate antibiotics. Ocular and oropharyngeal manifestations are rare but can occur with tularemia. Physicians should have a high clinical suspicion for tularemia in patients presenting with prolonged illness associated with either conjunctivitis or oral ulcers, especially in an endemic area. In children, aminoglycosides are the treatment of choice. Tetracyclines have higher rates of relapse, and fluoroquinolones, while effective, are not approved by the FDA.

## References

[1] (2024). Tularemia data and statistics. https://www.cdc.gov/tularemia/data-research/index.html.

[2] Snowden J, Stovall S (2011). Tularemia: retrospective review of 10 years' experience in Arkansas. Clin Pediatr (Phila).

[3] Bishop A, Wang HH, Donaldson TG (2023). Tularemia cases increase in the USA from 2011 through 2019. Curr Res Parasitol Vector Borne Dis.

[4] Quillin KP, Fornwalt BE, Potesta EL Jr, Nguyen CT (2019). Oropharyngeal tularemia: a case of ulcerative pharyngitis and necrotizing pyogranulomatous lymphadenitis. OTO Open.

[5] Lester Rothfeldt LK, Jacobs RF, Wheeler JG, Weinstein S, Haselow DT (2017). Variation in tularemia clinical manifestations-Arkansas, 2009-2013. Open Forum Infect Dis.

[6] Copur B, Surme S (2023). Water-borne oculoglandular tularemia: Two complicated cases and a review of the literature. Travel Med Infect Dis.

[7] Nemmour A, Bakri A, Fischer CA, Brand Y (2019). Paediatric oropharyngeal tularaemia requiring surgical intervention. BMJ Case Rep.

[8] Committee on Infectious Diseases (2024). Red Book: Report of the Committee on Infectious Diseases. 33rd ed..

[9] Wu HJ, Bostic TD, Horiuchi K, Kugeler KJ, Mead PS, Nelson CA (2024). Tularemia clinical manifestations, antimicrobial treatment, and outcomes: an analysis of US Surveillance Data, 2006-2021. Clin Infect Dis.

[10] Hassoun A, Spera R, Dunkel J (2006). Tularemia and once-daily gentamicin. Antimicrob Agents Chemother.

[11] Hofinger DM, Cardona L, Mertz GJ, Davis LE (2009). Tularemic meningitis in the United States. Arch Neurol.

[12] Barbaz M, Piau C, Tadie JM (2013). Rhombencephalitis caused by Francisella tularensis. J Clin Microbiol.

[13] Cross JT, Jacobs RF (1993). Tularemia: treatment failures with outpatient use of ceftriaxone. Clin Infect Dis.

